# Embankment seismic fragility assessment: A case study on Xi’an-Baoji expressway (China)

**DOI:** 10.1371/journal.pone.0246407

**Published:** 2021-02-05

**Authors:** Fa Che, Chao Yin, Xingkui Zhao, Zhinan Hu, Lu Sheng, Dong Liu

**Affiliations:** 1 Zibo Transportation Service Center, Zibo, China; 2 School of Civil and Architecture Engineering, Shandong University of Technology, Zibo, China; 3 Key Laboratory of Roads and Railway Engineering Safety Control (Shijiazhuang Tiedao University), Ministry of Education, Shijiazhuang, China; 4 State Key Laboratory of Mechanical Behavior and System Safety of Traffic Engineering Structures, Shijiazhuang Tiedao University, Shijiazhuang, China; 5 Shandong Dongtai Engineer Consulting Co., LTD., Zibo, China; 6 Laoling Branch of Dezhou Highway Development Center, Dezhou, China; Northeastern University, UNITED STATES

## Abstract

Although embankment seismic damages are very complex, there has been little seismic fragility research yet. Researches on seismic fragility of bridges, dams and reinforced concrete (RC) structures have achieved fruitful results, which can provide references for embankment seismic fragility assessment. Meanwhile, the influencing degrees of retaining structures, such as retaining walls on the embankment seismic performances are still unclear. The K1025+470 embankment of the Xi’an-Baoji expressway was selected as the research object, and the finite difference models of the embankment fill-soil foundation system and embankment fill-soil foundation-retaining wall system were established. The ground-motion records for Incremental Dynamic Analysis (IDA) were selected and the dynamic response analysis were conducted. Probabilistic Seismic Demand Analysis (PSDA) was used to deal with the IDA results and the seismic fragility curves were generated. Based on the assessment results, the influences of the retaining wall on the embankment seismic fragility were further verified. The research results show that regardless of which seismic damage parameter is considered or the presence or absence of the retaining wall, larger PGAs always correspond to higher probabilities of each seismic damage grade. Seismic damages to the embankment fill-soil foundation-retaining wall system are always lower than those of the embankment fill-soil foundation system under the same PGA actions, thus, the retaining wall can decrease the embankment seismic fragility significantly.

## 1 Introduction

Earthquakes are natural disasters occur in bursts and severely endanger people’s lives and properties [1]. The 1976 Tangshan earthquake in Hebei, China, the 1999 Chi-Chi earthquake in Taiwan, China, the 2008 Wenchuan earthquake in Sichuan, China, the 2010 earthquakes in Haiti and Chile, and the 2011 earthquake of the Pacific coast of Tōhoku in Japan and other previous violent earthquakes, highway embankments suffered from varying degrees of damages, which seriously disrupted the highway networks, becoming the “Gordian knots” for the whole earthquake relief work [2–5]. Embankment seismic fragility refers to the exceeding probabilities of different damage grades under earthquake actions, it can not only describe the relations between the ground-motion intensities and embankment damage grades, but also portray the embankment seismic performances [6–8]. The assessment results of the embankment seismic fragility can be used as the basis for embankment design and engineering fortification, suggesting reasonable options for improving highway seismic capacities [9–11].

Seismic fragility assessment started with the nuclear power plant, and reflected the results with the fragility curves and/or fragility matrixes [12–14]. A. Melani et al. [15] determined the financial risks on the basis of results of Incremental Dynamic Analysis (IDA) of reinforced concrete (RC) frames using nonlinear time history analyses with a suite of 20 ground motion records. Wang et al. [16] investigated the seismic fragility of arch dams using the dynamic damage analysis model of dam-reservoir-foundation systems, in which the radiation damping of semi-unbounded foundation rock, opening of contraction joints and damage cracking of dam concrete were taken into account. Ko and Yang [17] performed nonlinear finite element analyses using PLAXIS 2D for the seismic responses of sheet-pile wharves, and the modeling approach was verified to be satisfactory by simulating a 1-g scale-model shaking table test. Liu et al. [18] performed the seismic fragility analysis of recycled aggregate concrete (RAC) bridge columns with different recycled coarse aggregate (RCA) replacement ratios subjected to freeze-thaw cycles (FTCs) by the cloud analysis method. Yoon et al. [19] carried out nonlinear time history analyses for the pipeline considering soil-pipeline interaction represented by beam on nonlinear Winkler foundation model, and 12 ground motions were employed and four different analytical cases were considered to evaluate the effect of the uncertainty of soil parameters. Bao et al. [20] used both as-recorded and artificial seismic sequences as input to conduct the nonlinear dynamic analysis, and the effect of fault types of aftershocks on a mainshock-damaged containment was investigated in terms of the global response and local damage respectively. Chen et al. [21] used a small-scale shaking table model test to investigate the characteristics of the granular landslide deposits under influences of seismic wave, the results showed that vibration frequency significantly influenced the deposit shape. Pan et al. [22] employed the Latin hypercube sampling (LHS) technique to generate random samples of different uncertain parameters, and IDA was carried out to establish probabilistic seismic demand models (PSDMs) and develop fragility curves. Sainct et al. [23] proposed a methodology based on Support Vector Machine (SVM) coupled with an active learning algorithm to estimate fragility curves. Sarno and Pugliese [24] assessed the seismic fragility of typical existing RC structures subjected to earthquake sequences and various levels of corrosion, and a probabilistic approach and three different seismic intensity measures (IM) were proposed. Liang et al. [25] performed the approximate IDA and the slippage and sliding area ratio were chosen as the engineering demand parameters (EDPs), and different damage levels were identified by the slippage-based rule and sliding area ratio-based rule respectively according to their corresponding overall mean IDA curves. Kumar and Samanta [26] determined the log-normal variability functions by accounting for both the aleatory uncertainties and epistemic source uncertainties, and seismic fragility assessment was performed for different building categories in Patna, India. Ciano et al. [27] investigated the accuracy of fragility curves for an actual building struck by the 2016 Italian earthquake, and numerical analyses considering both linear and non-linear behavior of a multi-degree of freedom structural system subjected to this earthquake were performed. Ebrahimi et al. [28] used a number of effective techniques including LHS simulation, fuzzy set theory and *α*-cut approach to quantify the median of the collapse fragility curve as the fuzzy-random response. Ding et al. [29] conducted a series of shaking table tests of utility tunnels with and without a joint connection, the results showed that the structure without a joint connection presented a more significant acceleration response and horizontal soil pressure response than those with a joint connection.

Although embankment seismic damages are very complex, there has been little seismic fragility research yet. Researches on seismic fragility of bridges, dams and RC structures have achieved fruitful results, which can provide references for embankment seismic fragility assessment. Meanwhile, the influencing degrees of retaining structures, such as retaining walls on the embankment seismic performances are still unclear [7, 30]. In view of this, seismic fragility assessment of the K1025+470 embankment of the Xi’an-Baoji expressway was performed by IDA and Probabilistic Seismic Demand Analysis (PSDA), and fragility curves were generated. Based on the assessment results, the influences of the RC retaining wall on the embankment seismic fragility were further verified.

## 2 Methodology

Embankment seismic fragility assessment can be divided into empirical and theoretical methods. Empirical method is based on the field survey of the earthquake zone, and the empirical fragility curves are obtained through the integration of different ground-motion intensities and seismic damage grades [31–33]. Although the results of this method are accurate, its practical applicability is limited due to the following reasons [34–36]:

1. This method requires detailed ground-motion parameter distribution figures of the earthquake zone, but currently the figures are mainly obtained based on the existing attenuation laws combined with the monitoring site record values, their accuracy cannot completely meet the demand.
2. This method requires the damage grade figures of all the structures in the earthquake zone. On the one hand, determining the damage grade is highly subjective and the results are discrete; on the other hand, current damage surveys are mainly sampling surveys that do not cover all the structures in the earthquake zone.
3. This method can reflect the total seismic performances of one type of structure in the earthquake zone, but cannot reflect the specific seismic fragility characteristics of an monomer structure.

It is difficult to apply the empirical method over a wide range, particularly for highways and other linear structures [31, 35–37], therefore, the theoretical method was selected to perform the seismic fragility assessment of the K1025+470 embankment of the Xi’an-Baoji expressway, the main contents were as follows: (1) divide the embankment seismic damage grades, select the embankment seismic damage parameters and establish the relations between the seismic damage grades and seismic damage parameters; (2) establish the finite difference models of the embankment fill-soil foundation system and embankment fill-soil foundation-retaining wall system; (3) select the ground-motion records for IDA and clarify the dynamic response rules of the embankment; (4) determine the exceeding probabilities of different embankment damage grades under different PGAs and generate the fragility curves; (5) verify the influences of the retaining wall on the embankment seismic fragility.

## 3 Data preparation

Seismic damage grade classification method must be ascertained before assessing the embankment seismic fragility [38, 39]. In HAZUS99, bridges are classified into five states according to seismic performance, namely no damage, slight damage, moderate damage, severe damage and complete destruction [40]. In Japan, seismic damages to bridges, tunnels, slopes and highways are divided into five grades, namely severe, major, moderate, minor and very minor [41]. Referring to the above studies, embankment seismic damages were divided into five grades, namely basically intact, minor damage, moderate damage, severe damage and destroyed.

Little research has been reported on parameterizing the embankment seismic damages, but by analyzing the seismic damage parameters of other structures, it can be found that the selection of embankment seismic damage parameters should consider the following principles [42–45]:

1. Displacement is the most intuitive reflection of the seismic damages and the definitions of displacement parameters are simple, clear and easy to obtain, therefore, seismic damage parameters are mainly selected based on the displacement failure criterion.
2. Seismic damage parameters are comprehensive reflections of both local and overall seismic damages, they are also quantitative reflections of the degree of use-function reduction, therefore, more than one parameters often be chosen according to the actual situation.

The maximum lateral displacement rate (*ε*_*max*_) and maximum subsidence rate (*ζ*_*max*_) on the surface of the embankment were selected as the seismic damage parameters based on the displacement failure criterion. *ε*_*max*_ and *ζ*_*max*_ are defined in Eq (1).

$$\varepsilon_{max}=\frac{d_{max}}{D}{;}_{}\zeta_{max}=\frac{h_{max}}{H}$$ (1)

Where *d*_*max*_ is the maximum lateral displacement on the surface of the embankment, *D* is the width of the embankment fill, *h*_*max*_ is the maximum subsidence on the surface of the embankment, *H* is the maximum height of the embankment fill. According to the investigation results of the Wenchuan earthquake, embankments at the epicenter (Yingixiu town) suffered from the most severely damage, i.e. destroyed, and *ε*_*max*_ and *ζ*_*max*_ reached 1.059% and 1.210% respectively [46–48], therefore, considering *ε*_*max*_ = 1.0% and *ζ*_*max*_ = 1.2% as the critical values of “severe damage” or “destroyed” is reasonable. Besides, the critical values of *ε*_*max*_ and *ζ*_*max*_ among other embankment damage grades were further determined based on the equidistant classifying method [20, 38], as summarized in Table 1.

**Table 1:** Critical values of *ε*_*max*_ and *ζ*_*max*_.

Embankment seismic damage grades	Seismic damage parameters	
	*ε*_*max*_/%	*ζ*_*max*_/%
Basically intact	*ε*_*max*_<0.2	*ζ*_*max*_<0.2
Minor damage	0.2≤*ε*_*max*_<0.4	0.2≤*ζ*_*max*_<0.4
Moderate damage	0.4≤*ε*_*max*_<0.6	0.4≤*ζ*_*max*_<0.8
Severe damage	0.6≤*ε*_*max*_<1.0	0.8≤*ζ*_*max*_<1.2
Destroyed	*ε*_*max*_≥1.0	*ζ*_*max*_≥1.2

## 4 IDA of the embankment

### 4.1 Embankment model

**Fig 1:**
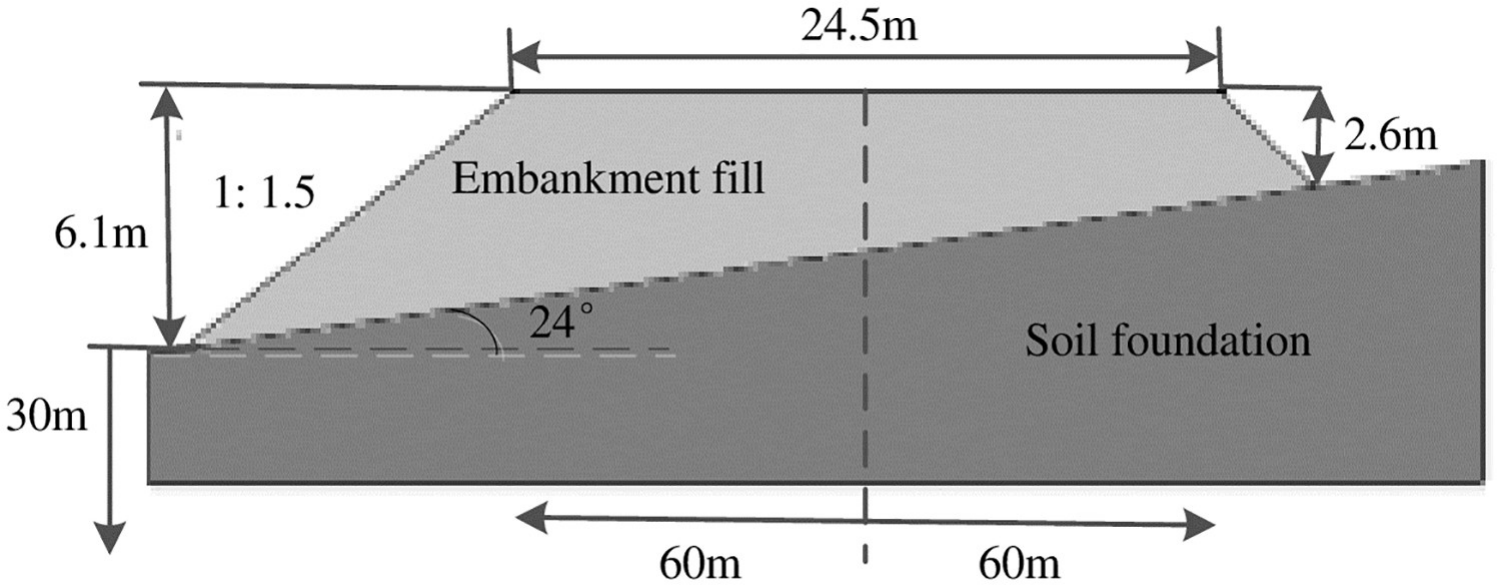
Finite difference model of the embankment fill-soil foundation system.

**Fig 2:**
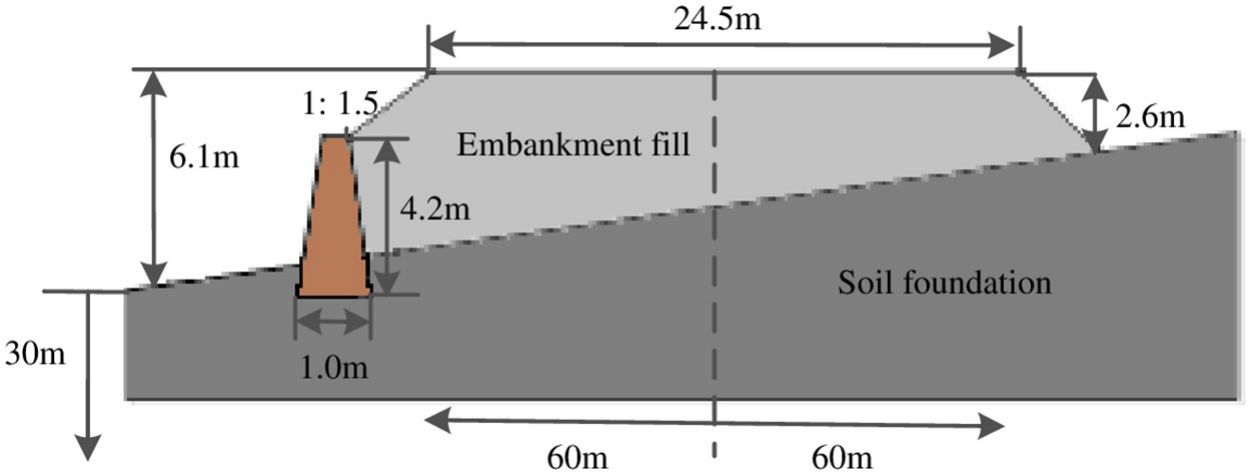
Finite difference model of the embankment fill-soil foundation-retaining wall system.

Xi’an-Baoji expressway is located in the Guanzhong plain where some sections are in the form of embankment [49, 50], the K1025+470 embankment was selected as the research object. By referencing on Castaldo et al. [51], a finite difference model of the embankment fill-soil foundation system was established via Flac software. The width of the embankment fill was 24.5m, the right slope was 2.6m high (minimum), the left slope was 6.1m high (maximum) and the slope ratio was 1: 1.5. The dip angle of the soil foundation was 24°, the thickness was 30m and the width was 120m. Among them, the vehicle loads had been converted to the thickness of the embankment fill according to the elastic layer theory [52], as shown in Fig 1. To verify the influences of the retaining wall on the embankment seismic capabilities, the existence of a RC retaining wall on the left slope of the embankment fill-soil foundation system was assumed, as shown in Fig 2.

An elastoplastic constitutive relation was employed in modeling the embankment fill and soil foundation, while an isotropic elastic constitutive relation was employed in modeling the retaining wall. The Mohr-Coulomb criterion was used as the yield criterion [51], and the mechanical properties of the embankment fill, soil foundation and retaining wall were determined, as shown in Table 2.

**Table 2:** Mechanical properties of the embankment fill, soil foundation and retaining wall.

Materials	Shear modulus	Density	Elastic modulus	Poisson’s ratio
Embankment fill	17.91MPa	1970.00kg/m^3^	48.00MPa	0.34
Soil foundation	15.67MPa	1630.00kg/m^3^	42.00MPa	0.34
Retaining wall	1282.05MPa	2300.00kg/m^3^	3000.00MPa	0.17
Materials	Bulk modulus	Internal friction angle	Cohesive force	
Embankment fill	50.00MPa	33.00°	34.00KPa	
Soil foundation	43.75MPa	28.00°	31.00KPa	
Retaining wall	1515.15MPa	--	--	

Under the actions of the ground-motions, the fundamental motion equation of the embankment fill-soil foundation system and embankment fill-soil foundation-retaining wall system is shown in Eq (2) [53].

$$M\ddot{u}+C\dot{u}+Ku=-MJ{\ddot{u}}_{g}$$ (2)

Where *M* refers to the total mass matrix containing the added vehicle mass, *C* refers to the total damping matrix, *K* refers to the total stiffness matrix, *J* refers to the indicator matrix of each seismic component, ${\ddot{u}}_{g}$ refers to the action of the ground-motion; $\ddot{u}$, $\dot{u}$ and *u* refer to the acceleration array, speed array and displacement array of the nodes respectively. The free-surface boundary was selected as the boundary conditions of the finite difference models, that was, the grids were generated on the model boundaries and the unbalanced forces of the free-surface grids were applied on the main grid boundaries [54]. The Rayleigh damping was adopted as the model damping, which simplified the damping matrix to a linear combination of the mass matrix and stiffness matrix [55]. The seismic fragility assessments were conducted on the embankment fill-soil foundation system and embankment fill-soil foundation-retaining wall system respectively, and the results were then compared [56, 57].

### 4.2 Determination of the ground-motion records

15 ground-motion records of 8 earthquakes provided by the United States Pacific Earthquake Engineering Research Center (PEER) were selected for IDA [58–60]. The epicentral distances (*E*_*d*_) of them are in the range of 10.9km to 50.9km, the magnitudes (*M*_*w*_) are in the range of 5.7 to 7.6 and the original PGA are in the range of 0.094g to 0.968g [61, 62], as shown in Table 3.

**Table 3:** Seismic ground-motion records.

No.	Earthquake	*E*_*d*_	*M*_*w*_	Original PGA	No.	Earthquake	*E*_*d*_	*M*_*w*_	Original PGA
1	Kobe_Japan	49.9km	6.9	0.094g	9	Cape Mendocino-2	18.5km	7.1	0.385g
2	Landers	50.9km	7.3	0.117g	10	Chalfont Valley-3	11.7km	6.0	0.447g
3	Bishop (Rnd Val)	19.0km	5.7	0.128g	11	Cape Mendocino-3	13.5km	7.1	0.591g
4	San Simeon_CA	38.0km	6.5	0.132g	12	Chi-Chi, Taiwan-1	26.0km	7.6	0.639g
5	Duzce_Turkey	34.3km	7.1	0.138g	13	Chi-Chi, Taiwan-2	18.8km	7.6	0.724g
6	Chalfont Valley-1	20.0km	6.0	0.143g	14	Chi-Chi, Taiwan-3	13.4km	7.6	0.821g
7	Cape Mendocino-1	33.8km	7.1	0.229g	15	Chi-Chi, Taiwan-4	10.9km	7.6	0.968g
8	Chalfont Valley-2	16.2km	6.0	0.248g					

**Fig 3:**
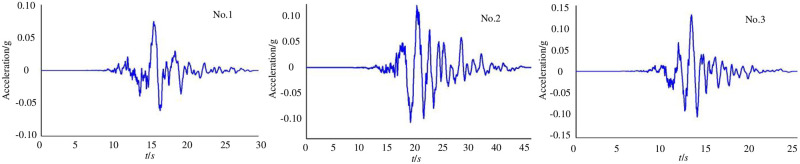
Acceleration time history curves of the No.1-No.3 ground-motion records.

Due to the limited space, the acceleration time history curves of the No.1-No.3 ground-motion records are listed in Fig 3.

In order to get the dynamic response characteristics of the embankment fill-soil foundation system and embankment fill-soil foundation-retaining wall system under different ground-motion intensities, the selected ground-motion records need to be adjusted to higher or lower intensity levels, that is, the amplitude modulation of ground-motion records. PGA of each ground-motion record was adjusted to 0.2g, 0.4g, 0.6g, 0.8g, 1.0g and 1.2g respectively, and obtained 90 ground- motion records [63, 64].

### 4.3 Dynamic response analysis

The 90 ground-motion records were input to the established models of the embankment fill-soil foundation system and embankment fill-soil foundation-retaining wall system for 180 dynamic response analysis. A total of 50 monitoring points were set up at every 0.5m on the surface of the embankment. The lateral displacement *d* and subsidence *h* at different monitoring points and different times as well as their mean values were recorded, and *ε*_*max*_, *ζ*_*max*_ and their mean values were calculated as shown in Tables 4 and 5.

**Table 4:** Dynamic response analysis results of the embankment fill-soil foundation system.

Serial number of the ground- motion records	0.2g		0.4g		0.6g		0.8g		1.0g		1.2g	
	*ε*_max_/%	*ζ*_*max*_/%	*ε*_max_/%	*ζ*_*max*_/%	*ε*_max_/%	*ζ*_*max*_/%	*ε*_max_/%	*ζ*_*max*_/%	*ε*_max_/%	*ζ*_*max*_/%	*ε*_max_/%	*ζ*_*max*_/%
1	0.1579	0.1812	0.4239	0.4876	0.5219	0.6389	0.8126	0.9937	1.3648	1.1854	1.4047	1.7159
2	0.2203	0.1438	0.3568	0.4471	0.5868	0.6977	0.7534	0.8543	1.2694	1.3148	1.5843	1.5342
3	0.1629	0.1398	0.3459	0.5128	0.5098	0.6018	0.8637	0.8875	1.1458	1.2991	1.4143	1.6487
4	0.2164	0.1716	0.4103	0.4095	0.5717	0.6844	0.8225	0.9109	1.3694	1.2675	1.5436	1.5846
5	0.1854	0.1379	0.3846	0.4167	0.5529	0.5079	0.7201	0.8456	1.1129	1.2834	1.5756	1.5241
6	0.1788	0.1812	0.4572	0.4891	0.5324	0.6047	0.7968	1.0077	1.2287	1.2037	1.5149	1.6008
7	0.1763	0.1251	0.3521	0.4359	0.6487	0.6387	0.7816	0.9768	1.1567	1.3651	1.5884	1.6017
8	0.1944	0.1723	0.3854	0.4765	0.5249	0.6916	0.8055	0.8391	1.3042	1.2513	1.4371	1.5418
9	0.1724	0.1454	0.4086	0.4123	0.6273	0.5721	0.7484	0.8746	1.1964	1.2789	1.6294	1.7309
10	0.1605	0.1948	0.3695	0.4896	0.5951	0.5948	0.7338	0.9427	1.2523	1.3811	1.5055	1.5281
11	0.1864	0.1335	0.4187	0.4312	0.5437	0.6989	0.8219	0.9009	1.0894	1.2846	1.5156	1.6807
12	0.2039	0.1861	0.4531	0.5195	0.5892	0.6251	0.7218	0.8248	1.1746	1.3718	1.5786	1.5438
13	0.1686	0.1565	0.4015	0.4047	0.5684	0.6984	0.6146	0.9986	1.1989	1.3064	1.4572	1.7158
14	0.1701	0.1779	0.4365	0.5464	0.5145	0.7478	0.7343	1.1001	1.3568	1.4049	1.3926	1.7872
15	0.1912	0.1248	0.3486	0.3751	0.6367	0.5495	0.7965	0.8064	1.1057	1.1285	1.6357	1.4597
Mean values	0.1830	0.1581	0.3968	0.4569	0.5683	0.6368	0.7685	0.9176	1.2217	1.2884	1.5185	1.6132

**Table 5:** Dynamic response analysis results of the embankment fill-soil foundation-retaining wall system.

Serial number of the ground- motion records	0.2g		0.4g		0.6g		0.8g		1.0g		1.2g	
	*ε*_max_/%	*ζ*_*max*_/%	*ε*_max_/%	*ζ*_*max*_/%	*ε*_max_/%	*ζ*_*max*_/%	*ε*_max_/%	*ζ*_*max*_/%	*ε*_max_/%	*ζ*_*max*_/%	*ε*_max_/%	*ζ*_*max*_/%
1	0.1192	0.0918	0.2771	0.2404	0.5276	0.5723	0.7542	0.8132	0.9679	1.1926	1.0257	1.1859
2	0.1097	0.1397	0.3281	0.3235	0.4151	0.4475	0.6343	0.6908	0.9287	1.0737	1.2459	1.2062
3	0.1412	0.1146	0.2932	0.2862	0.4292	0.5314	0.6537	0.7345	0.8782	0.9822	1.1732	1.2048
4	0.1266	0.1251	0.2906	0.2533	0.5387	0.4097	0.6443	0.6825	0.9103	1.0246	1.0362	1.3659
5	0.0958	0.0995	0.3393	0.2962	0.4262	0.5681	0.7016	0.7936	0.8583	1.0251	1.0842	1.4351
6	0.1481	0.1182	0.2806	0.2777	0.4384	0.4561	0.6732	0.7233	0.9437	0.8681	1.2235	1.2392
7	0.1026	0.1004	0.2414	0.2546	0.5007	0.5217	0.6497	0.7951	0.8831	1.0147	1.0267	1.4732
8	0.1408	0.1332	0.2995	0.2892	0.4116	0.5362	0.7316	0.8063	0.9059	0.9462	1.3006	1.1687
9	0.1129	0.1287	0.3056	0.2685	0.4282	0.4417	0.7096	0.7092	0.9635	1.0726	1.1876	1.2054
10	0.1487	0.1263	0.2414	0.2751	0.4997	0.4393	0.6472	0.7846	0.8847	1.0055	1.1046	1.3296
11	0.1243	0.1035	0.2298	0.2994	0.4258	0.5406	0.7351	0.7156	0.8462	1.1233	0.9258	1.4387
12	0.1074	0.0986	0.3037	0.2424	0.5081	0.5571	0.6943	0.6973	0.9517	1.0687	1.2533	1.2057
13	0.1286	0.0909	0.2834	0.2615	0.4679	0.4236	0.6625	0.8481	0.9028	1.1254	1.0932	1.3288
14	0.0919	0.1184	0.2587	0.2796	0.4136	0.5672	0.6234	0.8762	0.9716	0.9239	0.9953	1.4841
15	0.1489	0.1365	0.3316	0.2681	0.5517	0.4153	0.7768	0.6672	0.8346	0.9013	1.2782	1.1561
Mean values	0.1231	0.1150	0.2869	0.2744	0.4655	0.4952	0.6861	0.7558	0.9087	1.0232	1.1303	1.2952

**Fig 4:**
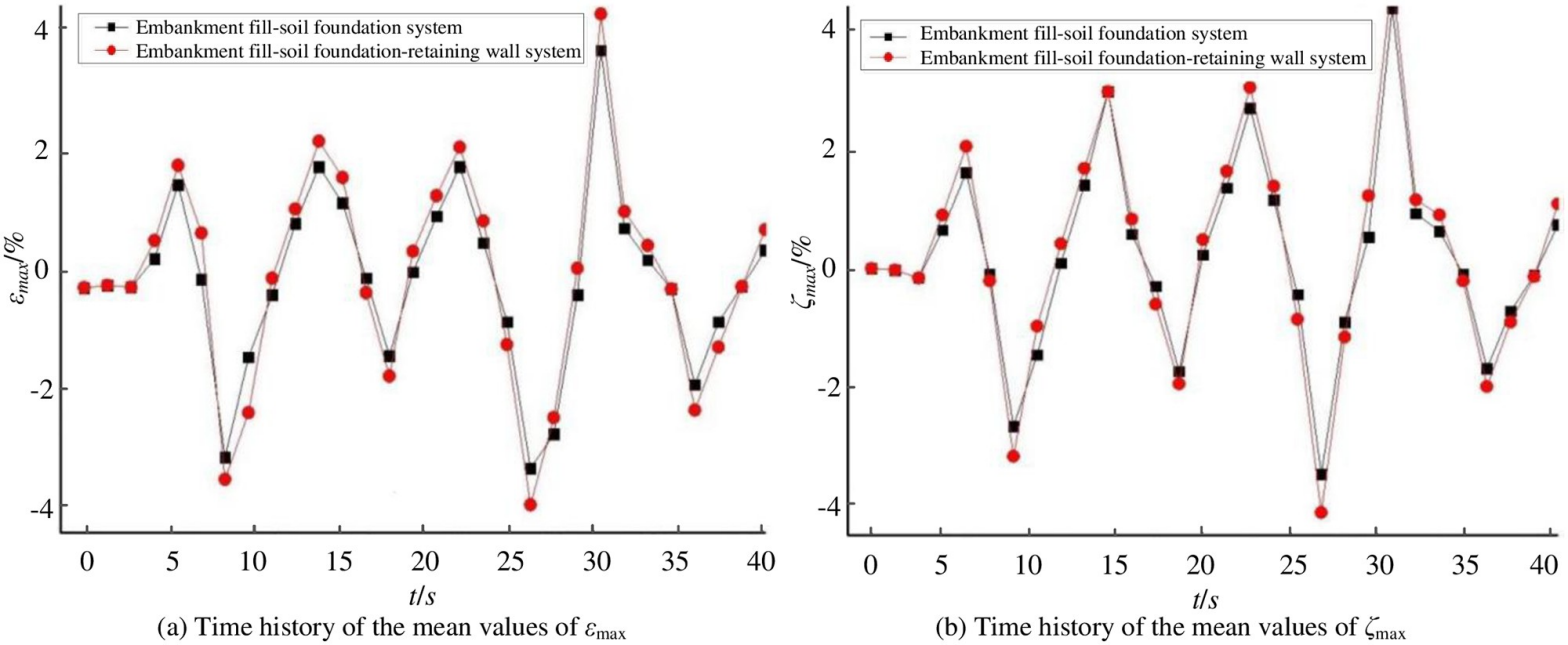
Time histories of the mean values of *ε*_max_ and *ζ*_max_ (positive values represent subsidence, negative values represent tilt).

Fig 4 illustrates the time histories of the mean values of *ε*_*max*_ and *ζ*_*max*_ on the monitoring point No.1 (left edge of the surface) of the embankment fill-soil foundation system and embankment fill-soil foundation-retaining wall system when PGA = 1.2*g*. It is evident from Fig 4 that the retaining wall reduces *ε*_*max*_ and *ζ*_*max*_ by 13.02% and 10.63% respectively.

### 4.4 IDA results

According to Karthik et al. [65], Alielahi and Moghadam [66] and Pang [67], *ε*_*max*_ and PGA follow the exponential relation, as shown in Eq (3).

$$\text{ln}\;\varepsilon_{max}=\text{ln}\;a_{1}\text{+}b_{1}\;\text{ln}\;\text{PGA}$$ (3)

Where *a*_1_ and *b*_1_ are estimated parameters. Similarly, *ζ*_*max*_ and PGA follow the relation shown in Eq (4).

$$\text{ln}\;\zeta_{max}=\text{ln}\;a_{2}\text{+}b_{2}\;\text{ln}\;\text{PGA}$$ (4)

**Fig 5:**
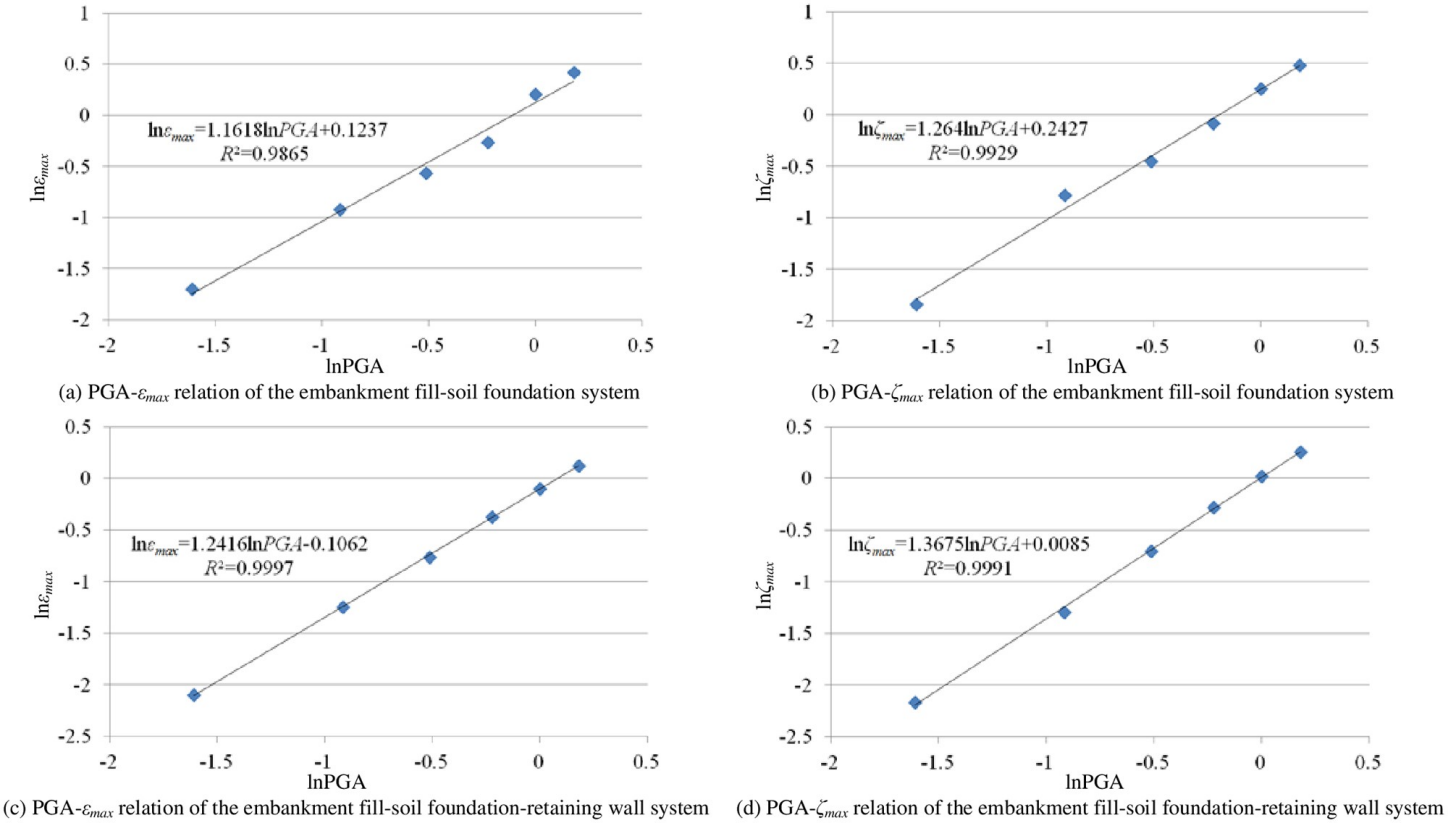
IDA results.

According to the dynamic response analysis results, regressions on *a*_1_, *b*_1_, *a*_2_ and *b*_2_ were performed and Fig 5 was obtained.

The relations between *ε*_*max*,_ *ζ*_*max*_ and PGA of the embankment fill-soil foundation system are shown in Eqs (5) and (6), and the relations between *ε*_*max*,_ *ζ*_*max*_ and PGA of the embankment fill-soil foundation-retaining wall system are shown in Eqs (7) and (8).

$$\varepsilon_{max}=1.1317{\text{PGA}}^{1.1618}.$$ (5)

$$\zeta_{max}=1.2747{\text{PGA}}^{1.264}$$ (6)

$$\varepsilon_{max}=0.8992{\text{PGA}}^{1.2416}.$$ (7)

$$\zeta_{max}=1.0085{\text{PGA}}^{1.3675}.$$ (8)

## 5 Embankment seismic fragility assessment results

### 5.1 Seismic fragility curves

Eqs (5)–(8) were substituted into the classical calculation equation of the seismic fragility to obtain Eqs (9)–(12), which Eqs (9) and (10) were the seismic fragility equations of the embankment fill-soil foundation system considering *ε*_*max*_ and *ζ*_*max*_ as the seismic damage parameters respectively, and Eqs (11) and (12) were the seismic fragility equations of the embankment fill-soil foundation-retaining wall system considering *ε*_*max*_ and *ζ*_*max*_ as the seismic damage parameters respectively [68–70].

$$P_{j}=\Phi (2\cdot \text{ln}\;(1.1317{\text{PGA}}^{1.1618}/S_{j}))$$ (9)

$$P_{j}=\Phi (2\cdot \text{ln}\;(1.2747{\text{PGA}}^{1.264}/S_{j}))$$ (10)

$$P_{j}=\Phi (2\cdot \text{ln}\;(0.8992{\text{PGA}}^{1.2416}/S_{j}))$$ (11)

$$P_{j}=\Phi (2\cdot \text{ln}\;(1.0085{\text{PGA}}^{1.3675}/S_{j}))$$ (12)

**Fig 6:**
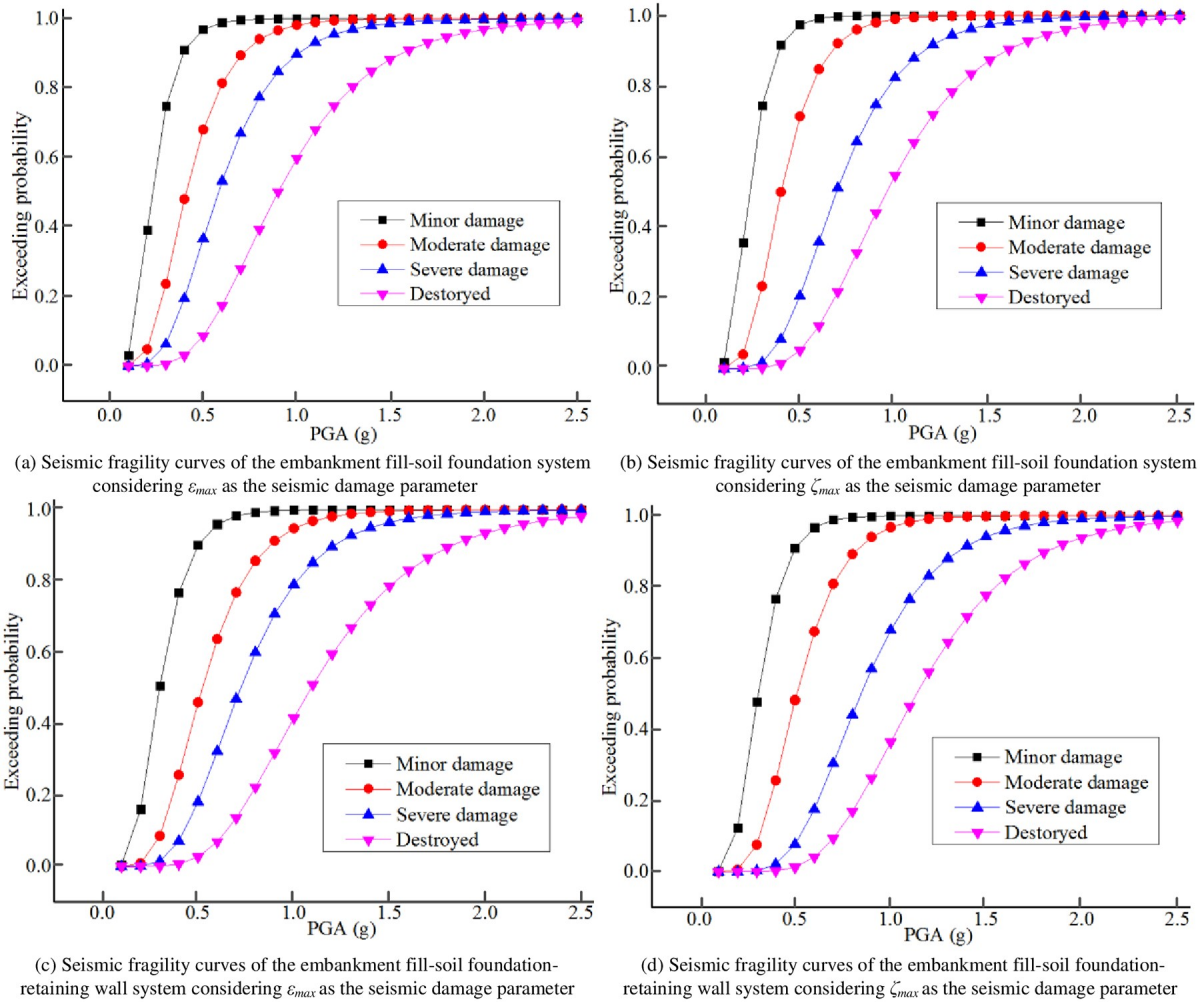
Seismic fragility curves.

Where *P*_*j*_ refers to the exceeding probability of the embankment seismic damage grade *j*, and *j* = 1 represents basically intact, *j* = 2 represents minor damage, *j* = 3 represents moderate damage, *j* = 4 represents severe damage, *j* = 5 represents destroyed; *S*_*j*_ refers to the structural performance level shown in Table 1, namely *S*_2_ = 0.20, *S*_3_ = 0.40, *S*_4_ = 0.60, *S*_5_ = 1.00 when considering *ε*_*max*_ as the seismic damage parameter, and *S*_2_ = 0.20, *S*_3_ = 0.40, *S*_4_ = 0.80, *S*_5_ = 1.20 when considering *ε*_*max*_ as the seismic damage parameter [71–73]. The embankment seismic fragility curves were generated according to Eqs (9)–(12), as shown in Fig 6.

### 5.2 Discussion

According to Fig 6, PGAs corresponding to each seismic damage grade with exceeding probabilities of 30%, 50% and 80% of the embankment fill-soil foundation system and embankment fill-soil foundation-retaining wall system were obtained, as summarized in Table 6.

**Table 6:** PGAs corresponding to each seismic damage grade with different exceeding probabilities.

Exceeding probabilities	Research objects	PGAs			
		Minor damage	Moderate damage	Severe damage	Destroyed
30%	Embankment fill-soil foundation system	0.1868g, 0.1947g	0.3393g, 0.3370g	0.4809g, 0.5831g	0.7465g, 0.8036g
	Embankment fill-soil foundation-retaining wall system	0.2504g, 0.2616g	0.4377g, 0.4343g	0.6067g, 0.7209g	0.9155g, 0.9697g
50%	Embankment fill-soil foundation system	0.2250g, 0.2310g	0.4085g, 0.3997g	0.5792g, 0.6917g	0.8990g, 0.9533g
	Embankment fill-soil foundation-retaining wall system	0.2980g, 0.3063g	0.5208g, 0.5085g	0.7219g, 0.8442g	1.0893g, 1.1356g
80%	Embankment fill-soil foundation system	0.3031g, 0.3039g	0.5505g, 0.5258g	0.7804g, 0.9099g	1.2114g, 1.2540g
	Embankment fill-soil foundation-retaining wall system	0.3939g, 0.3947g	0.6884g, 0.6552g	0.9543g, 1.0877g	1.4400g, 1.4630g

Note: the first data in each blank is the PGA when considering *ε*_*max*_ as the seismic damage parameter, while the second data is the PGA when considering *ζ*_*max*_ as the seismic damage parameter.

It is evident from Table 6 that although the coupling mechanism and mechanical process of the embankment fill, soil foundation and retaining wall under the earthquake actions are unclear, the embankment fill-soil foundation- retaining wall system always suffers from less damages than those of the embankment fill-soil foundation system. For example, for exceeding probabilities of 30%, 50% and 80%, the PGAs corresponding to the embankment fill-soil foundation-retaining wall system are 20.56%, 20.12% and 17.75% higher than those of the embankment fill-soil foundation system respectively when “destroyed” occurred, therefore, more serious seismic damages are less likely to happen to the embankment fill-soil foundation-retaining wall system. Similarly, the probabilities of each seismic damage grade of the embankment fill-soil foundation system and embankment fill-soil foundation-retaining wall system corresponding to different PGAs were calculated, as summarized in Table 7.

**Table 7:** Probabilities of each seismic damage grade corresponding to different PGAs.

Research objects	Seismic damage parameters	Seismic damage grades	Exceeding probabilities of each seismic damage grade corresponding to different PGAs											
			0.1g	0.2g	0.3g	0.4g	0.5g	0.6g	0.7g	0.8g	0.9g	1.0g	1.1g	1.2g
Embankment fill-soil foundation system	*ε*_*max*_	Basically intact	1.0000	1.0000	1.0000	1.0000	1.0000	1.0000	1.0000	1.0000	1.0000	1.0000	1.0000	1.0000
		Minor damage	0.0298	0.3923	0.7482	0.9094	0.9683	0.9887	0.9958	0.9984	0.9994	0.9997	0.9999	0.9999
		Moderate damage	0.0005	0.0485	0.2365	0.4804	0.6806	0.8141	0.8946	0.9408	0.9668	0.9812	0.9893	0.9939
		Severe damage	0.0000	0.0067	0.0632	0.1949	0.3664	0.5327	0.6701	0.7736	0.8471	0.8978	0.9320	0.9547
		Destroyed	0.0000	0.0002	0.0054	0.0299	0.0864	0.1737	0.2805	0.3932	0.5010	0.5977	0.6804	0.7489
	*ζ*_*max*_	Basically intact	1.0000	1.0000	1.0000	1.0000	1.0000	1.0000	1.0000	1.0000	1.0000	1.0000	1.0000	1.0000
		Minor damage	0.0171	0.3578	0.7456	0.9174	0.9745	0.9921	0.9975	0.9992	0.9994	0.9996	0.9998	0.9999
		Moderate damage	0.0002	0.0400	0.2340	0.5006	0.7142	0.8477	0.9217	0.9603	0.9799	0.9898	0.9948	0.9973
		Severe damage	0.0000	0.0009	0.0173	0.0831	0.2059	0.3596	0.5120	0.6434	0.7471	0.8242	0.8795	0.9181
		Destroyed	0.0000	0.0000	0.0017	0.0141	0.0514	0.1209	0.2174	0.3288	0.4421	0.5481	0.6412	0.7196
Embankment fill-soil foundation-retaining wall system	*ε*_*max*_	Basically intact	1.0000	1.0000	1.0000	1.0000	1.0000	1.0000	1.0000	1.0000	1.0000	1.0000	1.0000	1.0000
		Minor damage	0.0036	0.1610	0.5067	0.7676	0.9006	0.9589	0.9830	0.9929	0.9970	0.9987	0.9994	0.9997
		Moderate damage	0.0000	0.0087	0.0854	0.2562	0.4597	0.6374	0.7686	0.8568	0.9128	0.9474	0.9683	0.9809
		Severe damage	0.0000	0.0007	0.0146	0.0713	0.1809	0.3230	0.4695	0.6007	0.7080	0.7908	0.8522	0.8965
		Destroyed	0.0000	0.0000	0.0007	0.0064	0.0266	0.0693	0.1361	0.2217	0.3177	0.4159	0.5096	0.5949
	*ζ*_*max*_	Basically intact	1.0000	1.0000	1.0000	1.0000	1.0000	1.0000	1.0000	1.0000	1.0000	1.0000	1.0000	1.0000
		Minor damage	0.0011	0.1219	0.4772	0.7672	0.9099	0.9670	0.9881	0.9957	0.9984	0.9994	0.9998	0.9999
		Moderate damage	0.0000	0.0054	0.0745	0.2557	0.4816	0.6745	0.8089	0.8924	0.9408	0.9678	0.9826	0.9906
		Severe damage	0.0000	0.0000	0.0023	0.0205	0.0760	0.1752	0.3042	0.4415	0.5695	0.6784	0.7654	0.8319
		Destroyed	0.0000	0.0000	0.0001	0.0022	0.0124	0.0405	0.0929	0.1690	0.2624	0.3640	0.4653	0.5600

It is evident from Table 7 that regardless of which seismic damage parameter is considered or the presence or absence of the retaining wall, larger PGAs always correspond to higher probabilities of each seismic damage grade. For example, when PGA = 1.2g, the probabilities of the embankment fill-soil foundation system being “destroyed” are 11.12% and 18.46% respectively higher on average than those when PGA = 1.1g. On the other hand, the seismic damages to the embankment fill-soil foundation-retaining wall system are always lower than those of the embankment fill-soil foundation system under the same PGA actions. For example, when PGA = 1.2g, the probability of the embankment fill-soil foundation-retaining wall system being “destroyed” is 27.15% lower than that of the embankment fill-soil foundation system, thus, the retaining wall can decrease the embankment seismic fragility significantly.

## 6 Conclusions

1. Embankment seismic damages were divided into 5 grades, the maximum lateral displacement rate (*ε*_*max*_) and maximum subsidence rate (*ζ*_*max*_) on the surface of the embankment were selected as the seismic damage parameters. The K1025+470 embankment of the Xi’an-Baoji expressway was studied, the structure forms of the embankment fill-soil foundation system and embankment fill-soil foundation-retaining wall system were determined and the finite difference models were established via Flac software. The ground-motion records for IDA were selected and the dynamic response analysis were conducted. The PSDA was used to deal with the IDA results and generated the seismic fragility curves, the influences of the RC retaining wall on the embankment seismic fragility were further determined.
2. Regardless of which seismic damage parameter was considered or the presence or absence of the retaining wall, larger PGAs always correspond to higher probabilities of each seismic damage grade. Under the same PGA actions, the seismic damages to the embankment fill-soil foundation-retaining wall system are always lower than those of the embankment fill-soil foundation system, thus, the retaining wall can decrease the embankment seismic fragility significantly.
3. Although the embankment seismic fragility assessment was studied in this paper, the following problems still remained. First, while *ε*_*max*_ and *ζ*_*max*_ have feasibilities as the embankment seismic damage parameters, they still could not fully reflect the embankment seismic damage characteristics, selecting more reasonable parameters is yet to be studied. Second, there are multiple factors influencing the embankment seismic fragility, the influences of the retaining wall were quantitatively studied, the influences of other factors are yet to be studied. Third, design parameters of the embankment, such as the dip angle of the soil foundation may play an important role in the embankment seismic performance according to existing studies, therefore, sensitive analysis about dip angle should be performed in subsequence studies.

## Supporting information

S1 Data (XLSX)
